# Fatigue Life Analysis of Cyclone Separator Group Structure in a Reactor Device

**DOI:** 10.3390/ma18061214

**Published:** 2025-03-09

**Authors:** Yilian Shan, Jiye Sun, Xianglong Zhu, Yanhui Tian, Junyao Zhou, Yuzhe Ding, Benjie Ding, Jianke Du, Minghua Zhang

**Affiliations:** Zhejiang-Italy Joint Lab for Smart Materials and Advanced Structures, School of Mechanical Engineering and Mechanics, Ningbo University, Ningbo 315211, China

**Keywords:** reactor, S-N curve, numerical simulation, structural analysis, fatigue life

## Abstract

In the chlorination industry, the reactor is a crucial equipment in which the chlorination reaction takes place. However, when the reactor is subjected to complex conditions such as high temperatures (e.g., >200 °C) and high pressures (e.g., >10 MPa), its structural integrity is significantly compromised, leading to severe safety issues. In this study, the fatigue life of a reactor is analyzed, with particular focus on the fatigue behavior of the cyclone separator under varying working conditions, such as changes in the temperature, pressure, and chemical environment. Using finite element simulations under steady-state conditions and the S-N curve from fatigue testing, the fatigue life and potential weak points of the reactor under different amplitudes and vibration frequencies are analyzed and predicted. This analysis is conducted using a combined simulation approach with ABAQUS and Fe-Safe software, v 6.14. This work also considers the periodic vibrations at the base of the cyclone separator within the reactor. Fatigue simulations under different vibration conditions are performed to further assess the fatigue life of the reactor, providing a theoretical basis for the optimization of design and ensuring operational safety. In addition, the influence of welding zones on the fatigue life is discussed. The results indicate that the welding defects and stress concentration may cause the welded joint to become a critical weak point for fatigue failure. Therefore, the fatigue performance of the welding zone should be carefully considered during the design phase.

## 1. Introduction

Chlorination reactions play a crucial role in the chemical industry, as they enable the synthesis of chlorinated organic compounds and other key chemicals. As the core vessel for the chlorination reaction, the reactor’s structure and mechanical properties directly impact the stability and efficiency of the production process [1,2,3,4]. However, the complex operating environment of the reactor, characterized by factors such as temperature, pressure, and chemical corrosion, accelerates the aging of the reactor and significantly affects the material’s fatigue performance, particularly in vulnerable regions like the welding zones [5,6].

Over prolonged operation, fatigue damage gradually accumulates in the reactor materials, eventually leading to crack propagation and structural failure [7]. Therefore, fatigue life analysis has become an increasingly critical component of reactor design and operation, serving as a key factor in ensuring the reactor’s safety and long-term stability. With the growing understanding of fatigue damage mechanisms under varying operational conditions, fatigue life prediction methods have garnered significant attention. Fatigue testing and analysis of welded components can help elucidate the impact of welding defects and offer critical insights for predicting fatigue life. Such studies not only contribute to optimizing reactor design and material selection but also mitigate the risk of fatigue failure and improve the equipment reliability under extreme operating conditions, such as high temperatures, high pressures, and aggressive chemical environments.

Over the recent years, methodologies for fatigue life analysis have advanced significantly, evolving from traditional approaches based on empirical formulas to comprehensive prediction models that integrate numerical simulations and experimental data. Finite element analysis (FEA), as a core tool, facilitates the precise simulation of stress distribution and fatigue damage processes in reactors, particularly under complex working conditions involving nonlinear behavior and thermomechanical coupling. Specifically, under steady-state conditions, finite element simulations offer more accurate fatigue life predictions, providing a solid theoretical basis for structural optimization and safety assessment of reactors [8]. For example, Mohanty et al. [9] analyzed the heat transfer and fatigue life of a dual-loop pressurized water reactor through three-dimensional (3D) finite element modeling. Furthermore, they estimated the thermomechanical fatigue life of the reactor assembly based on a system-level finite element model, considering environmental correction factors. Using a fully mechanized fatigue modeling method, combined with experiments and the finite element method, Barua et al. [10] analyzed the cyclic stress of a 316 nuclear-grade stainless steel specimen under different fatigue loads over its entire life cycle. Niu et al. [11] proposed a fatigue life analysis model for reactor pressure vessels and explored the relationship between safety factors and structural fatigue reliability based on the stress–strength, load-life, and strength–damage interference theories.

Notably, the existing research on fatigue damage mechanisms primarily focuses on the material degradation processes of reactors under complex working conditions. When exposed to high temperatures, high pressures, and chemical media over extended periods, the fatigue behavior of reactor materials is typically characterized by crack propagation, plastic deformation, and yield. Through experimental research and numerical simulations, researchers have examined the fatigue characteristics of different materials (such as stainless steel and titanium alloys) under different working conditions, providing a theoretical foundation for fatigue life prediction. These studies focus on materials typically used in reactors, providing a theoretical foundation for fatigue life prediction. For instance, Gurumurthy et al. [12] investigated the creep–fatigue damage of process reactor components under high temperatures using an elastic analysis method and proposed an effective design method. Pohja et al. [13] addressed the creep–fatigue challenges in the design of the fourth-generation high-temperature reactors. Sarkar et al. [14] examined the cyclic stress–strain response and low cycle fatigue (LCF) behavior of 20MnMoNi55 pressure vessel steel, offering valuable insights for the life assessment and fracture mechanism analysis of pressure vessels. Taheri et al. [15] investigated the influence of welding residual stress and fatigue crack propagation threshold on crack stagnation under high-cycle thermal fluctuations, analyzing their role in crack propagation in reactor materials. Ma et al. [16] explored the impact of the shape and properties of Q345R welded joints on stress concentration and fatigue life, analyzing how various factors affect the fatigue behavior of welded joints.

The S-N curve, a classical tool for fatigue life prediction, is widely used in the fatigue analysis of materials. By illustrating the fatigue life of materials under different stress amplitudes, the S-N curve provides critical experimental data for fatigue analysis [17,18,19]. However, the S-N curve alone cannot fully reflect the fatigue behavior of materials under complex working conditions. Moreover, most existing studies focus on single working conditions, with a lack of systematic studies on the fatigue behavior of reactors exposed to complex environments, such as high temperature, high pressure, vibration, and chemical corrosion. Therefore, it is essential to investigate the fatigue behavior of reactors under the combined effects of multiple working conditions and to analyze the fatigue damage mechanisms in these complex environments. Such research can enable more accurate fatigue life predictions and safety evaluations, thereby supporting design optimization, material selection, and the long-term reliable operation of reactors.

To address this issue, using a domestic reactor as an example, this work combines finite element simulations and S-N curve analysis under steady-state conditions in ABAQUS and Fe-Safe to predict the fatigue life of the reactor under different amplitudes and vibration frequencies. The thermomechanical simulation in this study examines the 200–300 °C temperature range, mirroring industrial reactors’ operational parameters. This interval was derived from preliminary equipment design schematics and corroborated through empirical field measurements. Furthermore, the potential structural weaknesses under different conditions were explored. While existing studies have predominantly focused on reactor material degradation under single operational conditions, our study develops a systematic integration of multiphysics modeling and fatigue analysis, with a particular focus on the multifactorial interactions between corrosion, pressure, temperature, and vibration in reactor materials. This approach provides a more accurate basis for fatigue assessment, supporting the design optimization and safe operation of the reactor [20].

## 2. Fatigue Test

### 2.1. Specimen Preparation

The main material used in the cyclone separator assembly of the reactor is low carbon steel Q345R, which is one of the most widely used materials in pressure vessels and pipelines. Formerly known as 16MnR, the letter “R” indicates its suitability for pressure vessels. Q345R is commonly used to manufacture pressure vessels subjected to cyclic loading, making its fatigue life a critical consideration in design [21].

**Figure 1:**
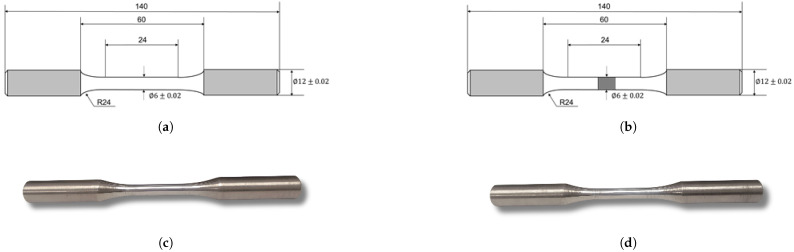
Q345R steel specimen and welding specimen for fatigue test. (**a**) Schematic depicting the dimensions of the Q345R steel specimen. (**b**) Schematic depicting the dimensions of the welded Q345R steel specimen. (**c**) Q345R steel specimen. (**d**) Welded Q345R steel specimen.

The standard tensile specimen for fatigue testing was made from a hot-rolled Q345R steel plate with a thickness of 15 mm. The specimen had a symmetrical structure, and its size adhered to the relevant standards [22]. The length of the specimen was 140 mm, and its central diameter was 6 mm. A transition arc with a radius of 24 mm was located in the center of the specimen. The weldment consisted of two welded pieces of Q345R round steel, with the overall size remaining unchanged. The welding wire used was Q235B, and the surface of the welded area was smooth and polished. Figure 1 presents the relevant details for both the specimen and the weldment sample.

To assess the impact of prolonged exposure to oxychlorination gases on the fatigue performance of Q345R steel, corrosion tests were performed by immersing specimens in 3.65% hydrochloric acid (HCl) for 200 h, 300 h, and 400 h. After immersion, residual acidic contaminants were neutralized using 3% sodium bicarbonate (${\mathrm{NaHCO}}_{3}$) solution, followed by extensive rinsing with deionized water to remove ionic residues. The specimens were vacuum-dried at 20 °C for 4 h until they were mass-stabilized, ensuring minimal moisture interference during subsequent fatigue characterization.

**Figure 2:**
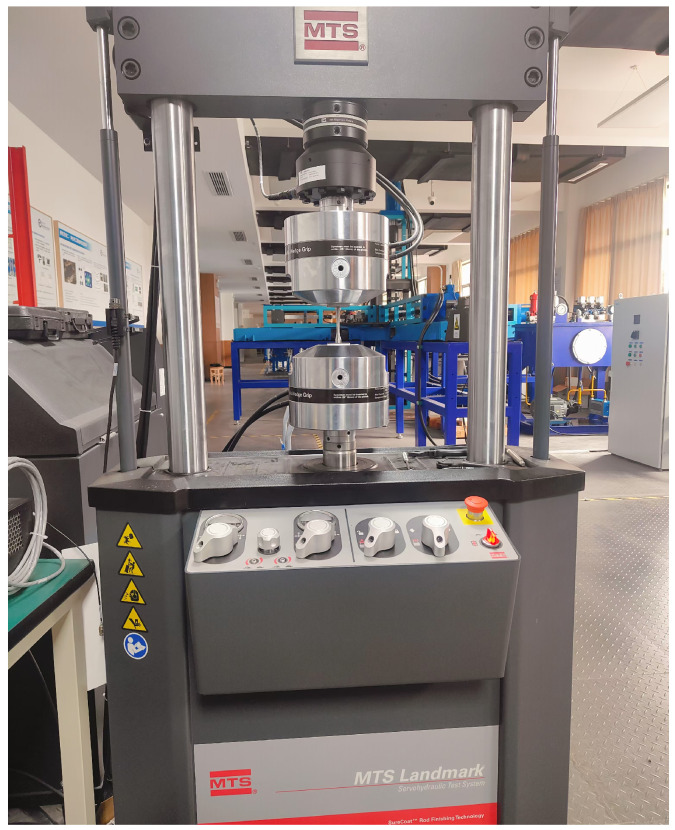
MTS 50 kN fatigue testing machine.

An MTS 50 kN fatigue testing machine was used to conduct fatigue tests at room temperature, as shown in Figure 2. The testing facility is equipped with computer control, and the applied load ranges from 3.5 kN to 11 kN. The system was designed to apply a symmetrical force to the sample in the coaxial configuration. The maximum allowable errors for the system were ±1% for the average static force and ±3% for the dynamic amplitude. The test was performed under tension–compression symmetrical loading conditions (R = −1) with a frequency of 10 Hz, and the fatigue life limit was set as $1\times 10^{7}$ cycles. A total of 30 samples were prepared for testing. Six sets of tests were conducted on both the original and welded samples, with each set involving three samples. The test ended when fatigue failure occurred or when the fatigue life reached or exceeded the set limit.

### 2.2. Test Result

**Figure 3:**
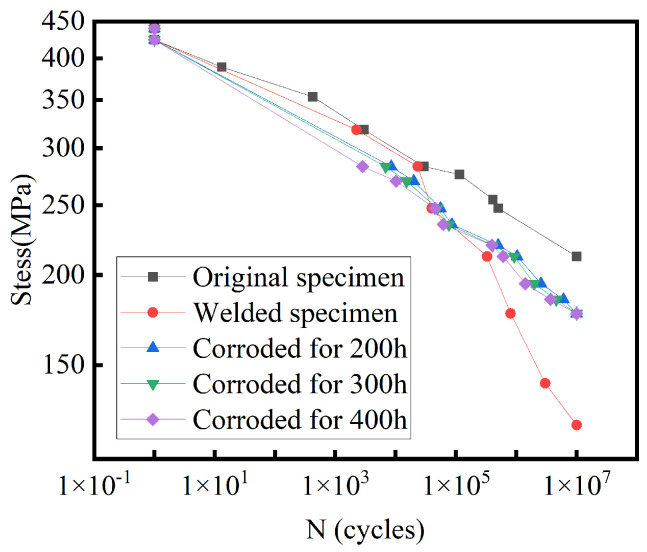
S-N curve of Q345R steel specimen, welded specimen, and corrosion-exposed steel specimens.

The S-N curve depicts the fatigue life (number of cycles) of a material at various stress levels. In this curve, “S” represents stress, usually the stress amplitude, and “N” represents the number of cycles, indicating the maximum number of load cycles the material can withstand at a given stress level. The S-N curve obtained from the test is shown in Figure 3. It is evident that the fatigue life of the Q345R steel specimen is significantly higher than that of the welded part of Q345R steel. This indicates that the fatigue performance of the original Q345R steel specimen is superior to that of the welded part. This difference suggests that the welding process may introduce stress concentration and welding defects, which reduce the fatigue performance of the welded area, thereby affecting the overall fatigue life of the structure.

The stress amplitude decreased with increasing cycle counts, which is consistent with fatigue damage accumulation over time. Welded specimens showed fewer cycles to failure than the base metal under identical stress due to stress concentrations and welding defects. This performance difference highlights welding’s critical impact on component fatigue life. Corrosion exposure reduced fatigue performance progressively with time (200–400 h HCl). Surface deterioration promoted crack initiation, while prolonged exposure caused microstructural weakening. Although corrosion degraded fatigue life, welding effects dominated due to microstructural changes.

## 3. Finite Element Model of Reactor

### 3.1. Geometric Model Parameters of Reactor Cylinder and Cyclone Separator

**Figure 4:**
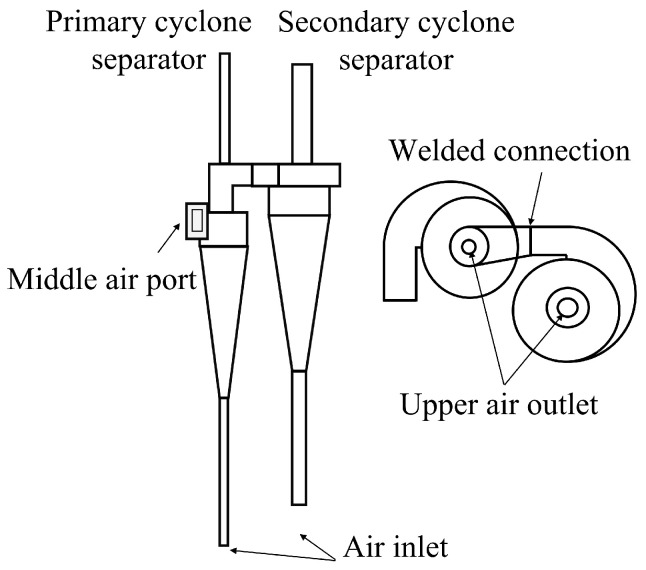
Structural diagram of cyclone separator.

The cyclone separator, a core component of oxychlorination reactors, is a dry gas–solid separation device that uses centrifugal force to effectively separate and capture dust particles from the rotating airflow. Its advantages include a simple structure, low energy consumption, and resistance to high temperatures and high pressures. The separator is primarily used for the preliminary separation of droplets and solid particles in the reaction products, thereby improving the purity of the gas [23,24]. The reactor studied in this work contains two sets of cyclone separators, with each consisting of a first-stage cyclone separator and a second-stage cyclone separator. Figure 4 shows a simplified structural diagram of the cyclone separator. The separator has a vertical volute cylinder structure, with the air outlet designed in three configurations: upper outlet, middle outlet, and lower inlet. This design allows the system to handle larger volumes of dusty gases while maintaining the same structural size, making it suitable for environments with high dust concentrations.

Additionally, this structure effectively reduces turbulence generated by the airflow inside the separator, minimizes wall wear, and prevents particle rebound during movement. The equipment’s nozzle is equipped with corresponding flanges, bolts, gaskets, and other connecting parts. To facilitate gas flow, the diameters of the upper and lower air outlets of the secondary cyclone separator are larger than those of the primary cyclone separator, and the middle air outlets of both the primary and secondary separators are welded together.

**Figure 5:**
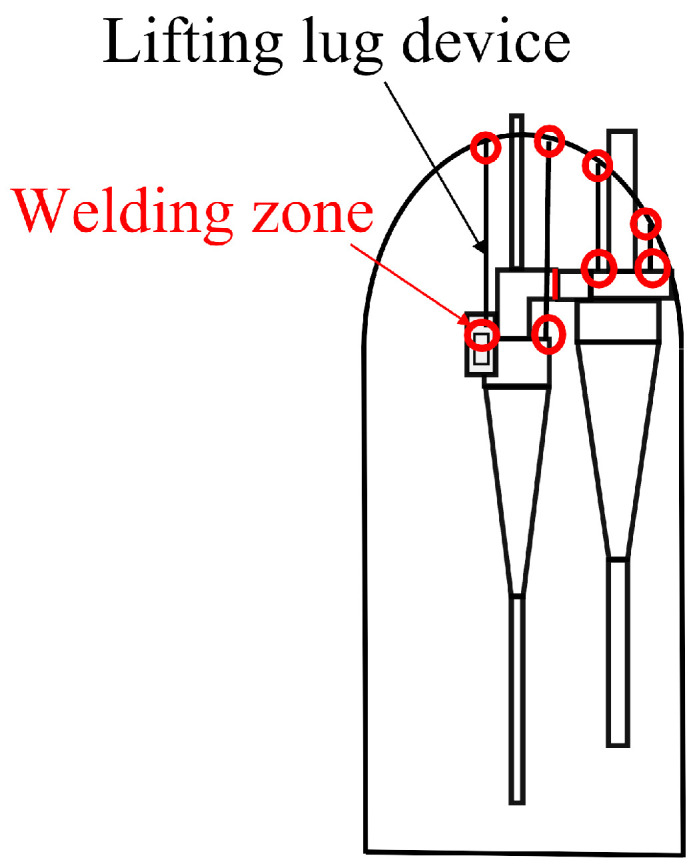
Schematic diagram of reactor structure.

Figure 5 shows a schematic of the reactor structure. The upper end of the reactor’s outer cylinder is welded to the gas outlet pipe, while the lower end is welded to the bottom skirt. Inside the reactor, a cyclone separator is installed, and the inner side of the upper head is equipped with a lifting lug for suspending the cyclone separator. The outlet at the top of the reactor is welded to the cyclone separator, and two sets of lifting lugs are used to support both the primary and secondary cyclone separators. The design and modeling of the cyclone separator and reactor structure were carried out using SolidWorks (version SW2023). Finite element analysis and stress simulations were performed using ABAQUS, which provided insights into the structural performance under various operating conditions.

### 3.2. Basic Assumptions in the Finite Element Model

The reactor has a complex structure and large volume, with numerous pipe coils and branch pipes. Given the two sets of symmetrical cyclone separators in the reactor, a 1/2 solid model is considered for simplification. Although there are constraints on the heat exchange tubes and the shell-side cylinder, the primary focus of the research is on the temperature field and stress field distribution across the entire reactor, distributor, and cyclone separator. Therefore, to streamline the modeling process, the following assumptions are made:

- The influence of thermometers, pressure gauges, and other components on the overall reactor performance is ignored.
- The influence of each nozzle, coil, and branch pipe assembly on the overall reactor performance is neglected.
- The internal components of the reactor are assumed to be closely integrated with the reactor cylinder and the head, and the contact gap between them is neglected.
- To simulate the welding assembly between components and prevent relative displacement, binding constraints are applied to the spiral air outlet, lifting lugs and shell, lifting lugs and cyclone separator, and the top outlet and shell of the cyclone separator.

In the numerical simulations, material properties such as thermal conductivity, specific heat capacity, Young’s modulus, and the thermal expansion coefficient are used to model the reactor components. These properties of Q345R low-alloy steel are summarized in Table 1.

### 3.3. Boundary Conditions and Mesh Division in the Finite Element Model Under Steady-State Conditions

**Figure 6:**
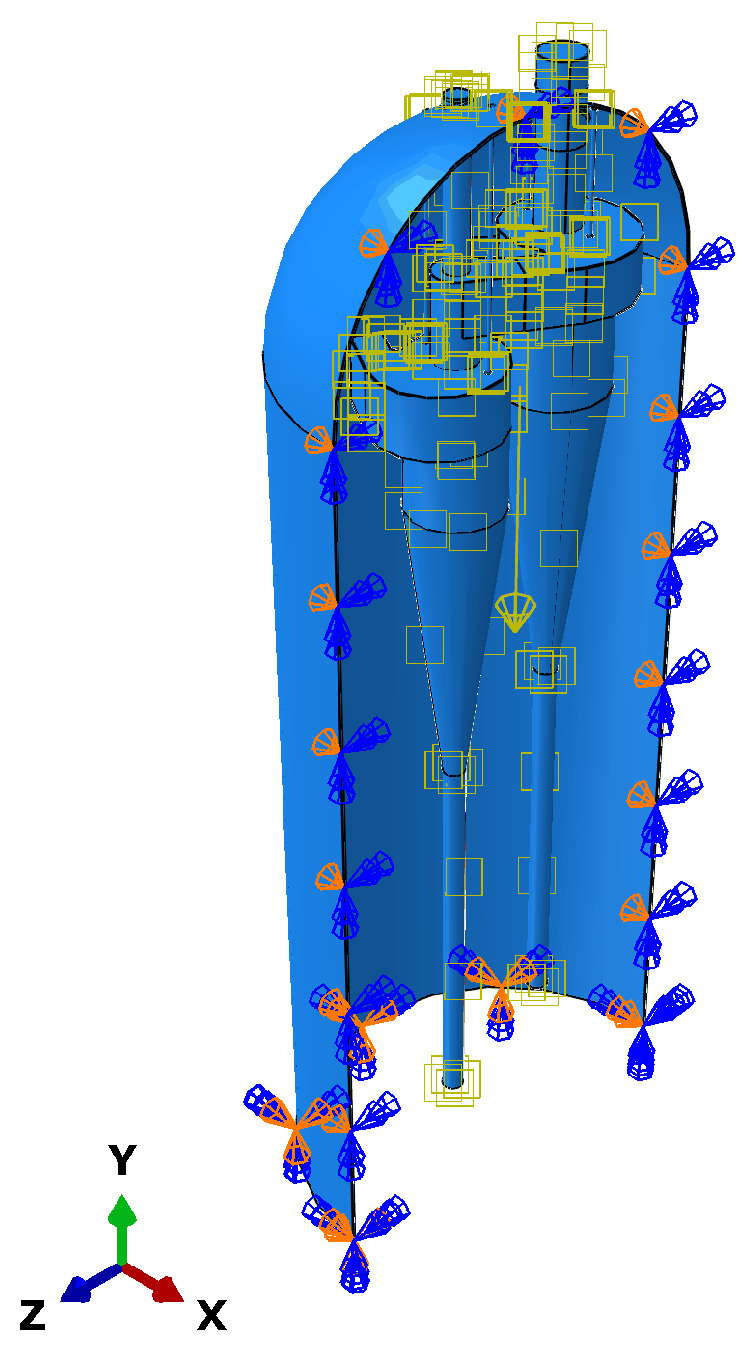
Boundary condition diagram.

Figure 6 illustrates the boundary conditions applied to the cyclone separator model. This figure shows the position of the support and the external force acting on the system, which is expressed in the 3D coordinate system. The boundary conditions of the model are set as follows:

- The degree of freedom in the normal direction of the symmetry plane is constrained.
- The bottom of the outer shell is set as a fixed boundary, with degree of freedom constraints applied in the X, Y, and Z directions.

This study primarily focuses on the influence of thermal stress on the reactor structure, so the effects of wind and snow loads, seismic load, and pipeline load are ignored in the calculations. The temperature boundary conditions are set as follows:

- The initial temperature of the distributor is set to 22 °C, and the temperature of the walls (including the upper head, cyclone separator, and other components) in contact with the working medium on the tube side is set to 227 °C.
- The temperature of the inner wall of the reactor linearly increases from 22 °C at the bottom to 227 °C at the top. The calculated temperature field is then imported into stress field analysis as grid temperatures. Since the model is symmetrical, only half of the model is considered in the stress analysis, with a symmetrical boundary condition applied at the symmetry surface.
- Considering the influence of the working medium, the air pressure is set to 0.43 MPa.

As shown in Table 2, the finite element analysis in this study was conducted using C3D10MT: a second-order thermomechanical coupled tetrahedral element, with each element consisting of four nodes and four degrees of freedom per node (UX, UY, UZ, TEMP). The model was discretized into a total of 144,706 elements and 278,123 nodes. The meshing approach used was an unstructured tetrahedral mesh with an adaptive size gradient, and the minimum mesh size was 0.1 mm, particularly at the connection between the cyclone separator and the lifting lug.

**Figure 7:**
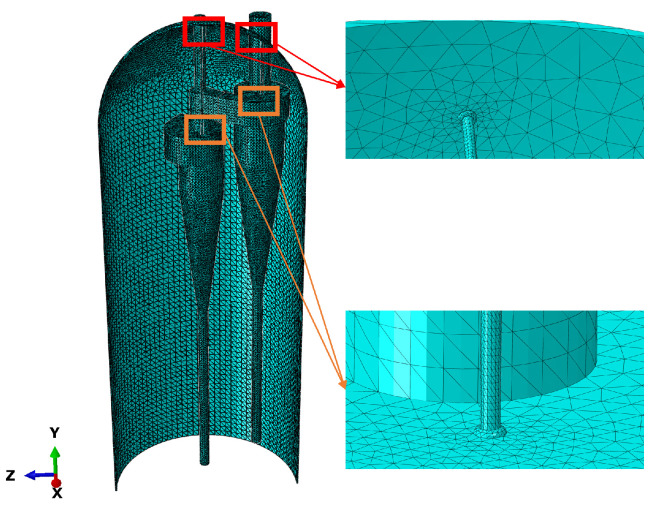
Reactor mesh diagram.

Figure 7 shows the mesh division of the reactor. Since the cyclone separator group of the reactor is connected to a large number of heat exchange pipes, thermometers, and pressure gauges, the structure is complex, and maximum stress typically occurs at the connection points between the components. Therefore, a dense grid was used for the connection areas between the components to ensure accurate stress analysis.

### 3.4. Calculation Results for Steady-State Operation of Reactor

**Figure 8:**
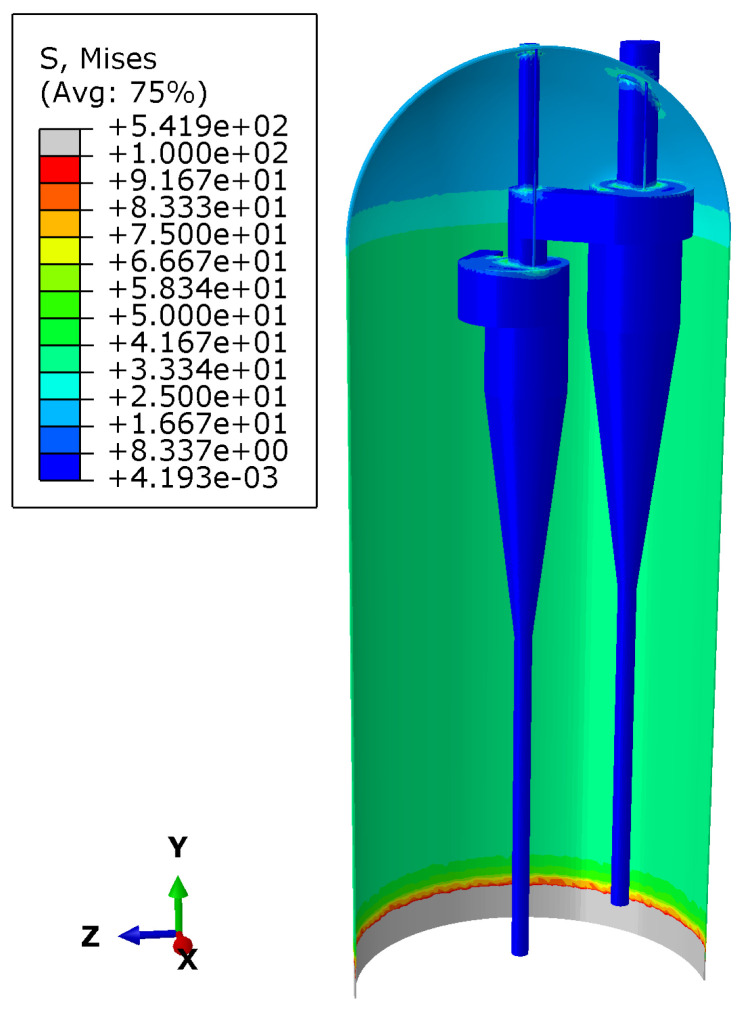
Stress distribution of the main structures during steady-state operation of the reactor (unit: MPa).

During steady-state operation, the reactor is subjected to pressure load (P) and temperature load (T). Figure 8 illustrates the stress distribution across the main structures of the reactor during the steady-state operation. The model simplifies the gas inlet connection area at the bottom of the reactor. Industrial waste gases, including carbon dioxide (${\mathrm{CO}}_{2}$), oxygen ($O_{2}$), ethylene glycol monomethyl ether (EGME), hydrogen chloride (HCL), and ethylene ($C_{2}H_{4}$), enter the reactor through the bottom inlet.

A significant temperature gradient existed at the bottom of the reactor cylinder, where the overall stress of the reactor reached a maximum of 541.9 MPa. The longitudinal stress distribution in the middle of the cylinder remained relatively flat, indicating a uniform stress distribution. However, a large stress gradient was observed in the upper cylinder area, especially near the cyclone separator and the connection between the two sets of lifting lugs. This suggests that these areas are potential stress concentration zones. When subjected to load, the connection between the reactor skirt and the cylinder is usually one of the high-stress areas [26].

**Figure 9:**
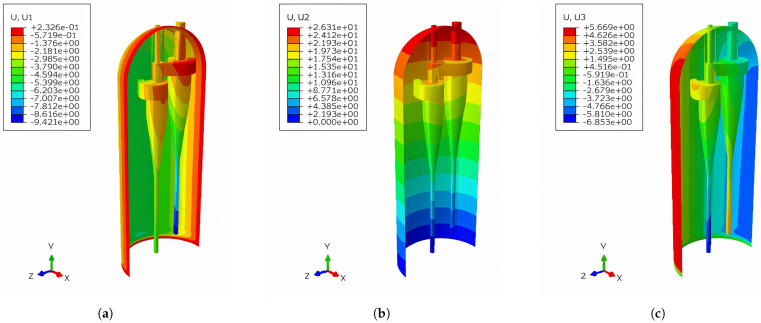
Displacement distribution of the structure during steady-state operation of the reactor (Unit: mm). (**a**) X-axis displacement distribution of the structure. (**b**) Y-axis displacement distribution of the structure. (**c**) Z-axis displacement distribution of the structure.

Figure 9 depicts the displacement distribution of the main structures of the reactor during steady-state operation. It can be seen that the deformation was primarily concentrated in the top outlet area of the cyclone separator. In addition, the cyclone separator above the support plate exhibited bending deformation. Meanwhile, the entire reactor cylinder showed a deformation characteristic of outward expansion. In terms of displacement, at the lowest end of the root of the cyclone separator, the X- and Z-axis displacements were approximately 10 mm, while the Y-axis displacement was around 1 mm.

### 3.5. Fatigue Analysis Flowchart

As the core component of the oxychlorination reactor, the working state of the cyclone separator directly impacts the safety of the equipment. When the frequency of the alternating loads approaches the natural frequency of the structural member, resonance may occur, leading to an amplified response and increasing the risk of structural fracture. In the oxychlorination reactor, the top of the upper cylinder supports the lifting load of the cyclone separator, and this vibration phenomenon further exacerbates the risk of fatigue failure.

**Figure 10:**
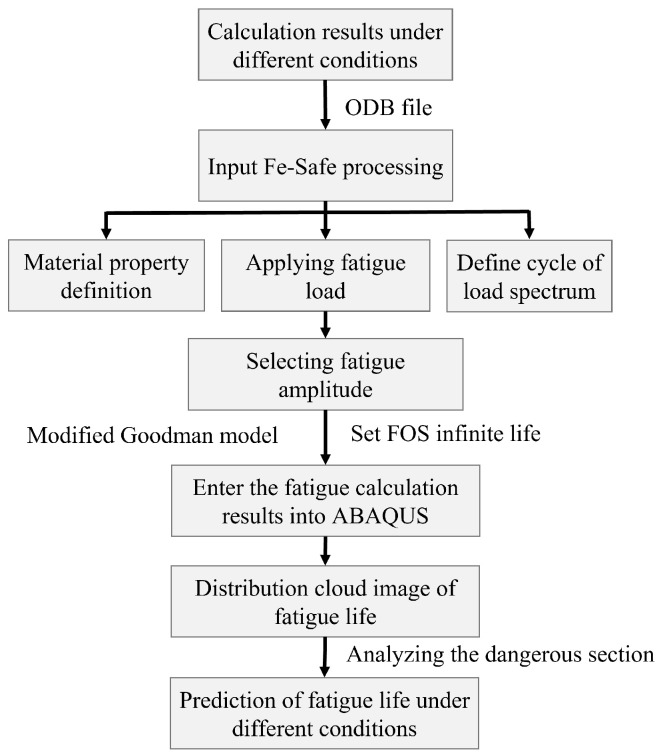
Fatigue analysis process of reactor [27].

To investigate the fatigue failure of the oxychlorination reactor structure during operation, particularly the stress and fatigue life at the root of the cyclone separator, ABAQUS and Fe-Safe software tools were used for a co-simulation fatigue analysis. The calculation process is outlined in Figure 10.

**Figure 11:**
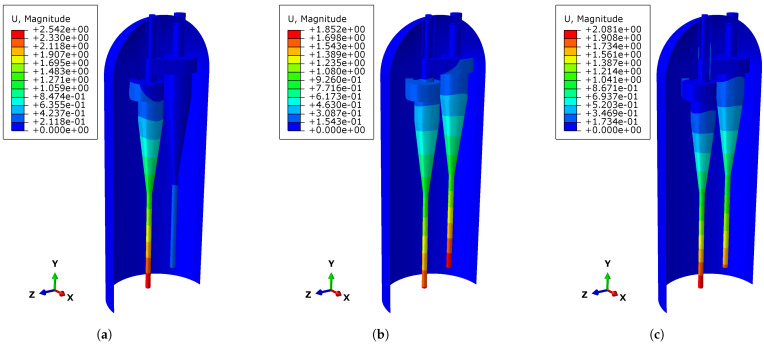
Vibration modes of the three frequencies (unit: mm). (**a**) Vibration mode of the reactor at a frequency of 1.2834 Hz. (**b**) Vibration mode of the reactor at a frequency of 2.4030 Hz. (**c**) Vibration mode of the reactor at a frequency of 2.8723 Hz.

The actual working conditions must be considered, particularly the vibration at the root of the cyclone separator inside the oxychlorination reactor, which may lead to resonance under certain conditions. Resonance is one of the common dangerous phenomena in engineering. Therefore, the natural vibration modes of the reactor were firstly simulated. Based on the calculation results, three frequency values, 1.2834, 2.4030, and 2.8723 Hz, were selected for the periodic vibration analysis. The corresponding vibration modes for these frequencies are shown in Figure 11. Next, periodic displacement was applied to the bottom node of the primary root of the cyclone separator, covering the X, Y, and Z directions. According to the steady-state calculation results, the displacement at the lowest end of the cyclone separator root was approximately 10 mm in both the X and Z directions and around 1 mm in the Y direction. Since the displacement in the horizontal X and Z directions is comparatively larger, the periodic displacement in the longitudinal Y direction has a more significant impact on the reactor. Therefore, during the analysis, the periodic displacement in the X and Z directions was set to 10 mm, while the displacement in the Y direction was set to 1 mm. The stress distribution of the reactor under different vibration frequencies was then calculated, and the result file was imported into Fe-Safe for fatigue analysis. After defining the fatigue characteristics and fatigue load spectrum, the fatigue life of the reactor under different vibration conditions was further analyzed to evaluate its reliability and safety during long-term operation, which typically spans several years.

In engineering applications, components subjected to cyclic loading typically experience an average load, and the presence of average tensile stress reduces the material’s fatigue limit. Therefore, the fatigue durability of materials is often assessed using the average stress correction method. Among the various methods, the Goodman model is widely used due to its simple formulation, high computational efficiency, and accuracy. As a result, it has become a standard stress correction method in engineering practice [28,29]. The Goodman model is expressed as follows:

(1) $$\sigma_{ar}=\frac{\sigma_{a}}{1-\frac{\sigma_{m}}{\sigma_{b}}}$$

where $\sigma_{ar}$ represents the corrected stress amplitude, and $\sigma_{b}$ is the tensile strength of the material. $\sigma_{m}$ is the actual average stress, and $\sigma_{a}$ is actual stress amplitude (unit: MPa). They are expressed as follows:

(2) $$\sigma_{a}=\frac{\sigma_{max}-\sigma_{min}}{2}=\frac{\sigma_{max}\left(1-R\right)}{2}$$

(3) $$\sigma_{m}=\frac{\sigma_{max}+\sigma_{min}}{2}=\frac{\sigma_{max}\left(1+R\right)}{2}$$

When the average stresss $\sigma_{m}=0$ (stress ratio R=1), the S-N curve is the basic curve. When $\sigma_{m}>0$, indicating the presence of average tensile stress, the S-N curve shifts downward. Conversely, when $\sigma_{m}<0$, implying the presence of average compressive stress, the S-N curve shifts upward, suggesting that the fatigue life of the material increases under the same stress amplitude [30].

On the S-N curve, the fatigue limit (or endurance limit) refers to the maximum stress level at which the material can withstand an infinite number of cyclic loads without experiencing fatigue failure [31]. Using the S-N fatigue curve, the fatigue life of the reactor under different frequencies and displacements can be calculated using the material data obtained from testing. In Fe-Safe, the durability limit of the material is typically set to $10^{7}$ cycles by default. When the material’s life exceeds $10^{7}$ cycles, it is considered to be in an infinite life state.

For infinite life analysis, the factor of safety (FOS) method is commonly employed. The calculation results are typically based on a reduction factor derived from the element stress, which is obtained from the set target life, also known as the “worst factor of safety”. Generally, the worst FOS ranges from a maximum of 2 to a minimum of 0.5 [32]. After setting the target life, the worst FOS is calculated. The output results can then be imported into ABAQUS to visualize the FOS distribution between the components.

By examining the FOS, the safe and fatigue conditions under different vibration frequencies and displacement amplitudes can be assessed as follows:

- If the FOS is less than 1, it indicates that the structure is unsafe at the current stress level, and the fatigue life under the current stress state will not reach the target life. This implies that the fatigue strength limit has been exceeded, posing a risk of failure.
- If the FOS is equal to 1, it means that the structure is operating at the fatigue strength limit under the current stress level, indicating a risk of failure.
- If the FOS is greater than 1, the structure is considered safe at the current stress level. The higher the FOS, the safer the structure, and the greater the load it can withstand.

## 4. Fatigue Analysis of Reactor Cylinder and Cyclone Separator

The design life of the reactor’s cyclone separator is typically 8 years, which corresponds to a total cycle time of $8\times 365\times 24\times 3600=252.288\times 10^{6}$ s. To assess the risk of vibration failure in the reactor’s natural vibration state, the frequencies of the first three modes—1.2834, 2.4030, and 2.8723 Hz—were selected for periodic vibration analysis. Accordingly, it is assumed that uninterrupted vibration occurs in the cyclone separator group of the reactor over the entire design cycle time. For each type of frequency, the target life is defined as follows:

(1) When the frequency is 1.2834 Hz, the target life is set to $3.23\times 10^{8}$ cycles.
(2) When the frequency is 2.4030 Hz, the target life is set to $6.06\times 10^{8}$ cycles.
(3) When the frequency is 2.8723 Hz, the target life is set to $7.24\times 10^{8}$ cycles.

As illustrated in Figure 10, the result files of the reactor under varying vibration conditions were imported into Fe-Safe to configure the fatigue analysis parameters, including load history, mean stress correction method, etc. The fatigue analysis results obtained from the calculations are presented in Table 3. It is evident that when the frequency is 2.4030 Hz and the displacement in the X direction (the normal direction of the reactor section) is 10 mm, the FOS is 0.997, which is lower than 1, and the fatigue life is $5.596\times 10^{8}$ cycles. When the frequency is 2.4030 Hz and the displacement in the Y direction (the longitudinal direction of the reactor) is 1 mm, the FOS is 0.884, again lower than 1, and the fatigue life is $2.162\times 10^{7}$ cycles. The fatigue curve specified in ASME BPVC [33] establishes the design criteria for reactor components, stipulating that the fatigue life within $10^{6}$–$10^{10}$ cycles. The derived safety factor (FOS) must always satisfy the minimum threshold established for welded parts in safety-critical applications, with a typical minimum requirement of FOS > 1 to ensure structural integrity and mitigate premature fatigue failure [34]. In these two scenarios, while the reactor’s cyclone separator assembly remains compliant with design specifications, it fails to achieve the target lifespan of $6.06\times 10^{8}$ cycles. In other cases, where periodic displacements are applied in different directions at varying frequencies, the FOS is generally higher than 1, indicating that the fatigue life under these conditions exceeds the target life, and the structure remains safe at the current stress levels.

The welding process can lead to stress concentration and welding defects, which significantly impact the fatigue performance of the welded area, thereby affecting the overall structural fatigue life. Therefore, when calculating the fatigue characteristics of the reactor, it is crucial to carefully consider the fatigue behavior of the welded area to improve the accuracy and reliability of the results. Based on the experimental S-N curve data for the simulated weldment, Table 4 presents the fatigue analysis results for the weldment. When periodic displacements were applied in different directions at varying frequencies, the FOS of the weldment was consistently found to be lower than 1, indicating that the fatigue life does not meet the target life at the current stress level, which suggests a risk of fatigue failure during long-term operation. Further analysis reveals that the stress concentration effect in the welded area has a significant influence on the fatigue performance, particularly under high stress and frequent cyclic loading conditions.

**Figure 12:**
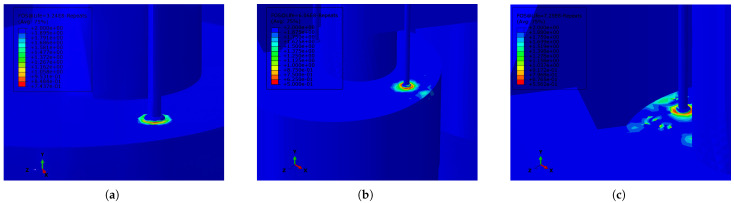
FOS distribution in the area above the cylinder in the reactor. (**a**) FOS distribution of the reactor (frequency: 1.2834 Hz; displacement: 10 mm along the X direction). (**b**) FOS distribution of the reactor (frequency: 2.4030 Hz; displacement: 10 mm along the X direction). (**c**) FOS distribution of the reactor (frequency: 2.8723 Hz; displacement: 10 mm along the X direction).

Figure 12 illustrates the FOS distribution for the cylindrical section of the reactor, as determined from the S-N curve for the welded joints in Q345R steel. Figure 12a–c demonstrate the spatial evolution of FOS in the reactor’s upper cylindrical region under harmonic displacements of 10 mm along the X direction at frequencies of 1.2834 Hz, 2.4030 Hz, and 2.8723 Hz. The FOS at the interface between the lifting lug and the cyclone separator is observed to be the lowest in the entire structure. Under various vibration conditions (such as variations in frequency and displacement magnitude), the welding area of the lifting lug supporting the cyclone separator exhibited the lowest fatigue life, confirming that this area is the most critical risk zone in the structure.

The corrosion test results and corresponding S-N curves for the material exposed to hydrochloric acid (HCl) for 200, 300, and 400 h were integrated into the reactor’s fatigue life calculations. The factor of safety (FOS) and fatigue life data, presented in Table 5, illustrate the effects of corrosion exposure on the material’s performance under cyclic loading in various directions. In the X direction, with increasing corrosion exposure, the FOS gradually decreases, indicating a diminished capacity of the material to withstand cyclic loading. Similarly, in the Y and Z directions, the fatigue life exhibits a corresponding decline with prolonged corrosion exposure, further evidencing the detrimental impact of corrosion on fatigue performance.The FOS values for specimens subjected to extended corrosion durations (300 h and 400 h) were typically lower than those for specimens exposed for 200 h, suggesting that prolonged corrosion exposure compromises the material’s fatigue resistance. Nevertheless, despite the reduction in fatigue performance due to corrosion, the material retained an acceptable fatigue life under specific loading conditions, notably in the Z direction, where the FOS remained relatively higher than in other directions.These findings underscore the critical importance of accounting for corrosion in the fatigue analysis of reactor components. Extended exposure to corrosive environments can significantly impair the material’s fatigue resistance, highlighting the necessity of judicious material selection and the implementation of protective measures in reactor design to ensure long-term operational safety and reliability.

## 5. Conclusions

Combined with the S-N curve and finite element simulation results under steady-state conditions, this study employed a coupled simulation approach using ABAQUS and Fe-Safe software to analyze the fatigue life and identify possible weak links in a reactor under varying vibration amplitudes and frequencies. The main findings are summarized as follows:

1. Fatigue performance of Q345R steel and welded joints: According to the S-N curve analysis, the fatigue life of Q345R steel specimens was found to be significantly higher than that of welded parts, highlighting the detrimental effects of welding on the fatigue performance. The difference indicated that the welding process might introduce stress concentration and welding defects, leading to reduced fatigue life in the welded regions, thereby impacting the overall structural integrity. The corrosion experiments conducted on Q345R steel specimens, which were exposed to hydrochloric acid for durations of 200 h, 300 h, and 400 h, revealed a significant reduction in fatigue life as exposure time increased. The S-N curves, which illustrate the relationship between stress amplitude and the number of cycles to failure, demonstrated a decline in fatigue performance. Specifically, longer corrosion exposure resulted in a more pronounced decrease in fatigue life, highlighting the detrimental impact of extended acid exposure on the material’s fatigue properties.
2. Deformation and stress distribution under steady-state conditions: Under steady-state operating conditions, the reactor cylinder exhibited outward expansion deformation, while the cyclone separator group mainly experienced upward displacement and overall bending deformation at its root area. The vibration frequencies were found to have a significant impact on the fatigue life of the reactor, particularly at the root of the cyclone separator. Stress distribution and fatigue analysis of key components like the cyclone separator and the condensing tube bundle helped identify the potential fatigue failure zones, such as the top outlet of the cyclone separator and the connection with the lifting lug.
3. Influence of displacement directions on fatigue life: Based on the S-N curve of the Q345R steel and simulation results, it was found that the displacements in the X, Y, and Z directions at different vibration frequencies significantly influenced the reactor’s fatigue life. Among them, the displacement in the Y direction had the greatest impact on the reactor’s fatigue performance. By contrast, the displacement in the X and Z directions hardly influenced the fatigue life of the cyclone separator. Additionally, fatigue analysis of the reactor using the S-N curve of Q345R steel welded joints showed a notable reduction in performance compared to the base material. The S-N curves obtained from specimens exposed to corrosion indicated that corrosion significantly accelerates fatigue degradation, resulting in a marked reduction in the fatigue life of welded joints. Moreover, the analysis highlighted specific regions within these welds. These areas are particularly susceptible to failure when subjected to both corrosive environments and cyclic loading.

Overall, this study provides useful insights into the fatigue behavior of reactors under complex operating conditions and offers practical guidance for improving the design and durability of key components, thereby ensuring the safe and reliable operation of industrial reactors (such as ones for oxychlorination process). The obtained data may be useful for evaluating the safety of reactor components. The identification of key stress concentration zones and potential failure areas, including the impact of corrosion, can facilitate the structural optimization of the equipment. These optimizations can reduce the stress concentration, enhance the reactor’s fatigue performance, and improve the service life of the equipment.

## Figures and Tables

**Table 1:** Thermal and mechanical properties of Q345R low-alloy steel [25].

Temperature (°C)	Thermal Conductivity ($W\cdot m^{-1}\cdot K^{-1}$)	Specific Heat Capacity ($J\cdot {\mathrm{kg}}^{-1}\cdot K^{-1}$)	Expansion Coefficient ($10^{-7}\cdot K^{-1}$)	Elasticity Modulus (GPa)	Poisson Ratio
20	52.5	460	73.5	206	0.27
100	51	490	84	203	0.28
200	48	520	110.5	195	0.29
300	44	560	123	188	0.31
400	39	600	132	181	0.33
500	36	680	137.5	167	0.24
700	33	700	140	167	0.24

**Table 2:** Mesh information.

Parameter	Setting Value
Element type	C3D10MT: Second-order thermomechanical coupled tetrahedral element
Node degrees of freedom	4/node (UX, UY, UZ, TEMP)
Total number of elements	144,706
Total number of nodes	278,123
Mesh type	Tetrahedral unstructured mesh (adaptive size gradient)
The minimum mesh size	0.1 mm (connection between cyclone separator and lifting lug)

**Table 3:** Fatigue life results of the reactor based on the S-N curve of Q345R steel.

Frequency (Hz)		X-Direction	Y-Direction	Z-Direction
1.2834	FOS	1.656	1.719	1.719
	Fatigue life (cycles)	-	-	-
2.40302	FOS	0.997	0.884	1.477
	Fatigue life (cycles)	$5.596\times 10^{8}$	$2.162\times 10^{7}$	-
2.8723	FOS	1.25	1.719	1.562
	Fatigue life (cycles)	-	-	-

**Table 4:** Fatigue life results based on the S-N curve of Q345R steel welded components.

Frequency (Hz)		X Direction	Y Direction	Z Direction
1.2834	FOS	0.744	0.781	0.7629
	Fatigue life (cycles)	$2.44\times 10^{7}$	$3.257\times 10^{7}$	$2.979\times 10^{7}$
2.40302	FOS	0.5	0.5	0.631
	Fatigue life (cycles)	679,747.562	376,173.125	9,991,432
2.8723	FOS	0.5	0.725	0.669
	Fatigue life (cycles)	5,214,660.25	$3.678\times 10^{7}$	$1.67\times 10^{7}$

**Table 5:** Fatigue life results based on the S-N curve of Q345R steel corroded components.

Frequency (Hz)	Direction		200 h	300 h	400 h
1.2834	X direction	FOS	1.156	1.078	1.031
		Fatigue life	-	-	-
	Y direction	FOS	1.188	1.109	1.062
		Fatigue life	-	-	-
	Z direction	FOS	1.18	1.102	1.055
		Fatigue life	-	-	-
2.40302	X direction	FOS	0.669	0.613	0.594
		Fatigue life	6,793,049.5	5,355,967.5	4,013,942.5
	Y direction	FOS	0.594	0.556	0.519
		Fatigue life	1,394,676.375	1,182,403.5	795,120.125
	Z direction	FOS	0.988	0.913	0.875
		Fatigue life	$5.251\times 10^{8}$	$4.116\times 10^{8}$	$1.651\times 10^{8}$
2.8723	X direction	FOS	0.831	0.781	0.744
		Fatigue life	$8.917\times 10^{7}$	$5.403\times 10^{7}$	$3.59\times 10^{7}$
	Y direction	FOS	1.125	1.039	0.994
		Fatigue life	-	-	$6.659\times 10^{8}$
	Z direction	FOS	1.031	0.95	0.913
		Fatigue life	-	$4.386\times 10^{8}$	$2.866\times 10^{8}$

Fatigue life unit: cycles.

## Data Availability

No new data were created or analyzed in this study.
